# Somaclonal Variation in *Chrysanthemum* × *morifolium* Protoplast Regenerants

**DOI:** 10.3389/fpls.2020.607171

**Published:** 2020-12-18

**Authors:** Tom Eeckhaut, Wouter Van Houtven, Silvia Bruznican, Leen Leus, Johan Van Huylenbroeck

**Affiliations:** Plant Sciences Unit, Flanders Research Institute for Agriculture, Fisheries and Food (ILVO), Melle, Belgium

**Keywords:** Asteraceae, flower traits, genome size, *in vitro*, ornamentals, phenotype, plant breeding, protoclonal variation

## Abstract

*Chrysanthemum* × *morifolium* protoplasts were isolated and regenerated to assess possible protoclonal variation in the regenerants. After a preliminary screening of the potential of different regeneration systems for protoplast regeneration, we produced a series of cut chrysanthemum ‘Arjuna’ leaf protoplast regenerants through liquid culture. Regenerants (54) were vegetatively propagated and grown under a commercial production system in 2 different seasons. All screened regenerants were significantly affected with regard to either flower number, flower size, flower weight, leaf weight, stalk weight, or plant size. A significant plant size reduction in 43/52 and 48/49 regenerants for both seasons was the most recorded effect. Also a reduction in flowering induction time up to 10 days, altered flower types and colors were observed. Differences between growing seasons were notable. Possible molecular backgrounds including genome size variation and commercial applications in breeding of chrysanthemum are discussed.

## Introduction

Plant protoplasts are plant cells that have been enzymatically or mechanically stripped of their cell walls. Theoretically, they have the potential to develop into any cell type present in mature plants, which is a sine qua non-condition for plant regeneration. The list of plant species that can be regenerated from protoplasts is, however, limited. The common approach to design a regeneration protocol is trial and error based, an exhaustive strategy given the number of parameters that is relevant for protoplast regeneration (reviewed by Davey et al., 2005). On top of that, the genotype dependency of regeneration, e.g., in *Apium graveolens* or *Kalanchoe blossfeldiana* (Bruznican et al., 2017; Cui et al., 2019) limits the design of widely applicable protocols in many crops. Innovative approaches as, e.g., electrical stimulation or adding surfactants or antibiotics have significantly contributed to the regeneration of protoplasts isolated from recalcitrant genotypes or species (Davey et al., 2005; Eeckhaut et al., 2013). This has increased the application potential of protoplasts for plant breeding. Historically, the main protoplast based breeding strategy is intra-or interspecific somatic fusion (Johnson and Veilleux, 2001), which bypasses barriers typically related to sexual hybridization like sterility or early seed abortion. On the other hand, in addition to a regeneration protocol, fusion methodology needs to be designed for protoplast fusion, and in the event of asymmetric fusion fragmentation tools like irradiation, microprotoplast isolation and organelle silencing are equally required. Yet, to introduce novelties within a commercial crop through somaclonal variation during protoplast culture, a regeneration protocol suffices.

Somaclonal variation (Larkin and Scowcroft, 1981) can result from base deletion or substitution, changes in chromosome number, chromosome rearrangements, or changes in epigenetic marks, like hyper- or hypomethylation (Krishna et al., 2016). Undifferentiated cells like protoplasts or calli seem to be particularly prone to somaclonal variation compared to axillary buds and meristems (Bairu et al., 2011; Krishna et al., 2016). Stressful situations linked to the generation of free radicals typically contribute to the recovery of somaclones. The current status of (epi)genetic changes occurring *in vitro* and the triggering stress factors has been described by Bednarek and Orlowska (2019). Protoplasting, including wounding, cell wall degradation and subsequent culture of isolated cells, is an extreme example of stress (Smulders and de Klerk, 2011).

Somaclones may be identified with morphological, physiological, molecular or cytogenetic tools (Bairu et al., 2011). Fossi et al. (2019) performed an in-depth sequencing of potato protoclones, that were characterized by large scale aneuploidy and chromosome segment deletions and duplications. Moreover, different dosage profiles in leaves of the same protoclone indicated persistent instability. These frequent and multiple genomic changes as demonstrated through sequencing are very likely linked to the phenotypic alterations in vegetatively propagated crops.

Breeders have therefore exploited somaclonal variation in a number of commercial crops as reviewed by Krishna et al. (2016). The potential of protoplasts as explant type has been demonstrated in a number of studies. Rice protoplast regenerants were aberrant in plant height, flag leaf length and width, panicle length, number of primary branches, number of spikelets per panicle and number of seeds per panicle (Lee et al., 1999). Carrot protoplasts selected with *Alternaria radicina* fungal culture filtrate yielded individuals with decreased susceptibility toward the pathogen (Grzebelus et al., 2013). As for ornamentals, regenerants from mesophyll and cell suspension derived *Dianthus* protoplasts exhibited variations like an abnormal morphology with a decreased chromosome number, precocious flowering and vigorous growth with tetraploidy (Shiba and Mii, 2005). Winkelmann et al. (2008) detected altered ploidy levels, malformed flowers and delayed flowering after multiple vegetative meristem formation in *Cyclamen* protoplast regenerants. The degree of variation depended on the genotype and the time period between protoplast isolation and plant regeneration.

*Chrysanthemum* × *morifolium* Ramat (2*n* = 6*x* = 54, fam. Asteraceae) has a genome size between 17.95 and 19.16 pg/2C (Miler et al., 2020). It is used either as cut flower or pot plant and is one of the world’s most popular floricultural plants (Spaargaren and Van Geest, 2018). Breeding and research have mainly focused on flowering earliness, winter hardiness, flower colors, size and form, plant habit, day neutrality, and self-incompatibility (Anderson, 2006). Nowadays, intergeneric breeding aims to improve aphid and drought resistance, to modify growth habit or floret morphology; interspecific breeding focuses on improving hardiness and classical intraspecific breeding strives for optimized plant shape and growth, postharvest performance and pest and disease resistance. Development of DNA markers associated with these traits and the compilation of linkage maps is compromised because *Chrysanthemum* is a polysomic hexaploid (Spaargaren and Van Geest, 2018).

In addition to crossbreeding, spontaneous and induced mutagenesis is a major mainstay of innovation in *Chrysanthemum* (Datta, 2013). Within the contemporary pot and cut chrysanthemum assortments more half of the cultivars are sports. Tissue culture can be used to facilitate mutagenesis and genetic transformation (Nasri et al., 2018), but also to induce somaclonal variation (Jain, 2001), by enabling regeneration from a single affected cell. *In vitro* somaclonal variation can thus maximize the variation achieved by natural or induced sporting. Its potential is demonstrated by a number of case studies. Variations in ploidy levels and flower color in *Chrysanthemum* plants originating from shoot tip callus or petals (Bush et al., 1976) and after culture of capitula and stems (Miyazaki and Tashiro, 1978) were described. Flower color variation through culture of petal and leaf explants was obtained (Sutter and Langhans, 1981; Khalid et al., 1989; Miler and Zalewska, 2014). Ray floret regenerants showed variation in vegetative growth and flowering (Malaure et al., 1991). The explant type is an important parameter with regard to somaclonal variation; shoot-tip explant regenerants are more true-to-type than regenerants from adventitious buds (Zalewska et al., 2007). Tissue culture can be combined with irradiation, a traditional *Chrysanthemum* breeding strategy, and this approach enhanced the number of color mutants as well as the spectrum of color mutations (Okamura et al., 2015).

So far protoplast culture in *Chrysanthemum* is almost unexploited. Several parameters are of significant importance with regard to *Chrysanthemum* protoplast regeneration to either plants or microcalli: genotype (Sauvadet et al., 1990), cell density and complex additives (Fujii and Shimizu, 1990), starting material (Lindsay and Ledger, 1993), leaf age and osmoticum type (Endo et al., 1997), conditioned medium (Zhou et al., 2005), and culture type (Eeckhaut and Van Huylenbroeck, 2011).

Contemporary tools offer prospects for a more rationalized approach of protoplast culture, e.g., by studying reactive oxygen species, DNA (de)condensation and phytohormone levels (Eeckhaut et al., 2013). Yet, this has not led to a generally applicable *Chrysanthemum* protoplast regeneration protocol, although shoot regeneration can be achieved for particular genotypes (Adedeji et al., 2020).

**FIGURE 1:**
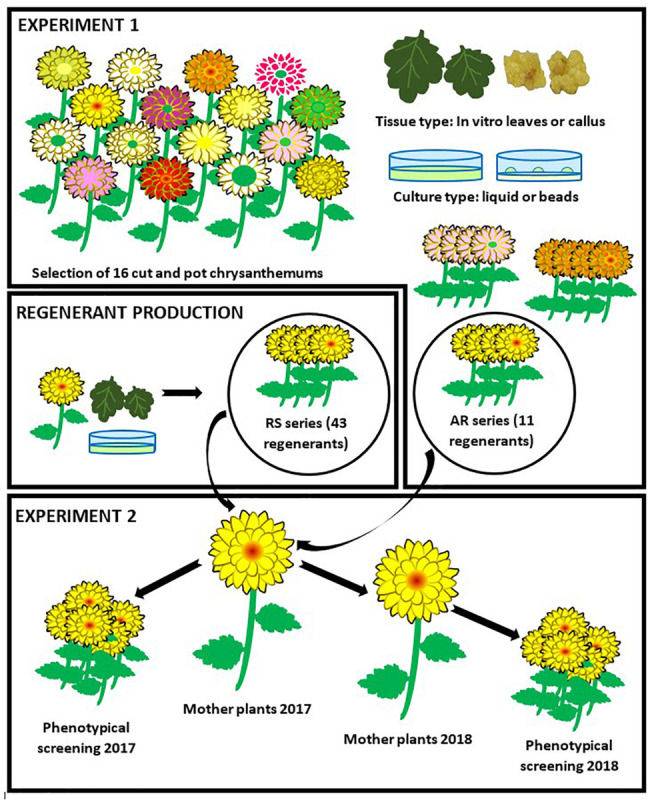
Experimental setup. In experiment 1, 16 cut and pot *Chrysanthemum* × *morifolium* cultivars were selected and the regeneration potential of protoplasts from 2 different tissues was tested in 2 different culture systems. A regeneration system (cultivar × tissue type × culture type) was selected. The regenerants of the selected cultivar were further referred to as the AR-series. Following, a larger regenerant batch (RS series) was produced through the selected system. In experiment 2, mother plants were grown from all AR and RS regenerants and used for cutting production. These cuttings were compared to the original cultivar through assessment of phenotypic parameters. For a second and independent assessment, new mother plants were produced from the old ones.

The goals of our research were (i) to select a performant *Chrysanthemum* × *morifolium* protoplast regeneration system (ii) to quantify and characterize possible protoclonal variation of *Chrysanthemum* × *morifolium* protoplast regenerants obtained through this system and (iii) to evaluate the stability of protoclonal variation during commercial production. In a first experiment the protoplast regeneration potential of a diverse collection of pot and cut chrysanthemum cultivars was tested and a single regenerative genotype and regeneration system were selected. In a second experiment, we regenerated plants from protoplasts of the selected cultivar and measured a series of morphological parameters in 2 independent screening trials to quantify and characterize protoclonal variation within the regenerant pool (Figure 1).

## Materials and Methods

### Experiment 1

Cut chrysanthemum cultivars ‘Euro Speedy,’ ‘Lindi White,’ ‘Oscar,’ ‘Snowflake,’ ‘Pointer,’ ‘Arjuna,’ ‘Chironne,’ ‘Felsina,’ ‘Ice Star’ (provided by Dekker Chrysanten, Netherlands) and pot chrysanthemum cultivars ‘Mostri White,’ ‘Passarela Purple,’ ‘Vigorelli Violet,’ ‘Odet Yellow’ (provided by Gediflora, Belgium), ‘Golden Surfer’ and ‘Le Bonheur’ (provided by VIVES Roeselare, Belgium) were used. Shoot tip (3 ± 0.5 cm) stock cultures were maintained in Meli-jars (23 ± 2°C, 16 h photoperiod, 40 μmol m^–2^ s^–1^ photosynthetic active radiation supplied by OSRAM L36 W/31 cool white fluorescent lamps). The stock medium was composed of Murashige and Skoog (1962) salts (MSS), Kao and Michayluk (1975) vitamins (KMV), 20 g/l sucrose, 2 mg/l glycine, 1 mg/l kinetin and 0.01 mg/l NAA (pH 6.2) and solidified with 6 g/l MC29 agar (Lab M Limited, United Kingdom). Cultures were refreshed every 8 weeks. As an alternative protoplast source material, callus was induced on *in vitro* leaves of a random selection of the aforementioned cultivars by placing them on callus induction media in the dark (abaxial side down) and making incisions perpendicular to the main leaf vein. The callus induction media were composed of MSS, KMV, 30 g/l sucrose, 2 mg/l glycine (6 g/l MC29 agar, pH 5.8) and supplemented with phytohormones based on callogenesis in previous experiments. For ‘Arjuna’ this medium was enriched with 0.5 mg/l NAA and 0.5 mg/l BA; for ‘Mostri White,’ ‘Passarela Purple,’ ‘Vigorelli Violet,’ ‘Odet yellow,’ and ‘Golden Surfer’ 3 mg/l BA and 0.2 mg/l IAA were added.

Leaf protoplasts of ‘Euro Speedy,’ ‘Lindi White,’ ‘Oscar,’ ‘Snowflake,’ ‘Pointer,’ ‘Arjuna,’ ‘Chironne,’ ‘Felsina,’ ‘Ice Star,’ ‘Golden Surfer,’ and ‘Le Bonheur’ were isolated according to Eeckhaut and Van Huylenbroeck (2011). Briefly, 500 mg fresh material from young *in vitro* leaves was digested for 16 h by an enzyme mixture of 0.5% cellulase Onozuka R-10 (Duchefa Biochemie BV, Netherlands), 0.3% macerase R-10 (Duchefa Biochemie BV, Netherlands) and 0.1% driselase from Basidiomycetes spp. (Sigma-Aldrich, Belgium); subsequently, the protoplasts were purified by filtering through a 100 μm nylon sieve and centrifugation (100 g, 10 min). Callus protoplasts of ‘Mostri White,’ ‘Passarela Purple,’ ‘Vigorelli Violet,’ ‘Odet Yellow,’ ‘Golden Surfer,’ and ‘Arjuna’ were isolated using a modified enzyme mixture of 1.5% cellulase, 0.5% macerase and 0.1% driselase and starting from 1,000 mg callus.

Callus protoplasts as well as leaf protoplasts were cultured based on the liquid culture protocol of Eeckhaut and Van Huylenbroeck (2011). Briefly, during the first week protoplasts were cultured in ½ MSS (without NH_4_NO_3_), KMV, 0.4 M mannitol, 10 g/l sucrose, 500 mg/l glutamine, 150 mg/l inositol, 1 g/l MES buffer, 2 mg/l NAA and 0.5 mg/l BA (pH 5.6), in Petri dishes (Φ = 3.5 cm, 1 ml medium, start density 10^5^ protoplasts/ml). The cultures were weekly refreshed by centrifuging (100 g, 10 min) and resuspended in new medium while diluting two- to fivefold depending on protoplast division rates, and transferred to Petri dishes, Φ = 5.5 cm. After 1 week, inositol was omitted from the medium and NAA was reduced to 0.5 mg/l. After 2, 3, and 4 weeks, mannitol concentrations were reduced to 0.32, 0.21, and 0.11 M, respectively. The climate room conditions were as aforementioned for stock cultures, except for a 12 h photoperiod. Five to 6 weeks after protoplast isolation, developed calli were transferred to semisolid medium [1/2 MSS, KMV, 10 g/l sucrose, 2 mg/l glycine, 0.5 mg/l BA, 0.02 mg/l NAA, 4 g/l Phytagel^TM^ (Sigma), pH 6.2], in Petri dishes (30–40 calli per dish, Φ = 9 cm, 25 ml medium per dish) and grown in the dark. Two weeks later, calli were put on regeneration medium (MSS, KMV, 20 g/l sucrose, 2 mg/l glycine, 0.1 mg/l TDZ, 6 g/l MC29 agar, pH 6.2 at a density of 15 calli per Petri dish, Φ = 9 cm). Cultures were kept in the dark and refreshed every 4 weeks until shoot formation.

In addition, ‘Golden Surfer,’ ‘Le Bonheur,’ and ‘Arjuna’ leaf protoplasts were cultured in LMPA beads. To this end, protoplasts suspended in liquid culture medium at a density of 2.10^5^ pp/ml were 1:1 mixed with LMPA medium [MS macrosalts without NH_4_NO_3_ and KNO_3_, Heller (1953) microsalts and KCl, 18.35 mg/l FeNa-EDTA, Morel and Wetmore (1951) vitamins, 100 mg/l inositol, 750 mg/l glutamine, 10 g/l sucrose, 60 g/l mannitol, 0.5 mg/l NAA, 0.5 mg/l BA, 5 g/l LMPA, pH 5.5], according to Deryckere et al. (2012). Five 50 μl beads were pipetted in a Petri dish (Φ = 5.5 cm) and 5 ml liquid culture medium was added. The liquid medium was weekly replaced by new culture medium. All liquid media were identical to the ones used for the liquid culture. Five to 6 weeks after protoplast isolation, calli were dissected from the beads. Culture on semisolid medium and regeneration medium was as aforementioned.

After a single multiplication cycle on stock medium, all regenerants were moved to rhizogenesis medium (stock medium without phytohormones). After rooting, agar was carefully removed, and plantlets (single shoots) were acclimatized for 12 weeks in plastic seedling trays, in a fog unit of the greenhouse (18–20°C, 16 h photoperiod, 95% RV) and subsequently grown in peat substrate (Structural 2C, pH 5.0–6.5, EC 450 μS/cm, 1.25 kg/mł 14N:16P:18K, Snebbout n.v.) in 11–21 cm diameter containers. A concise, non-quantitative morphological screening was performed upon plant flowering, including general plant, leaf and flower morphology and flowering time.

### Experiment 2

#### Plant Material

A series of 43 ‘Arjuna’ protoplast regenerants (RS1-RS12, RS14-RS19, RS24-RS35, RS37-RS43, and RS45-RS50) was produced, acclimatized, vegetatively multiplied and grown under commercial greenhouse circumstances (Dekker Chrysanten, Hensbroek, Netherlands) in order to quantify their protoclonal variation. All plants were regenerated from ‘Arjuna’ leaf protoplasts in liquid culture, meticulously following all culture parameters as selected in the first experiment. Moreover, also 11 regenerants from the first experiment derived from ‘Arjuna’ callus protoplasts (AR2, AR3, AR7, AR8, AR9, AR11, AR12, AR13, AR15, AR16, and AR18) were screened. These 54 protoplast regenerants were used as mother stock plants; parameters were measured in 2 independent assessments (PAs) during 2 different seasons in 2 consecutive years (2017–2018). For the second PA, new mother plants (1 per regenerant) had been cloned from the old ones. In both PAs, cuttings from plants that had not been cultured *in vitro* were included as a control, henceforth referred to as V-C (*in vivo* control). In the second PA, a supplementary control T-C (*in vitro* control) was included by acclimatizing *in vitro* multiplied *Chrysanthemum* cuttings of ‘Arjuna’ that had not been regenerated from protoplasts.

#### Growth Conditions

The two PAs were performed in August 2017 and May 2018. Culture practices were identical in both PAs. Per mother plant, 20 cuttings of 10 cm were taken when possible, rooted after dipping in Rhizopon Chryzotop^®^ 0.25% IBA and moved to a greenhouse under long day conditions (21,5 h light) where they were planted in 4 rows at a plant density of 45 plants m^–2^. After 9 days, short day conditions (11 h light) were applied for 10 weeks until flowering. During this induction period the average daily temperature varied between 21.7 and 23.3°C; the minimal and maximal temperatures were 16.3 and 33.4°C, respectively. The light intensity at plant height was kept between 100 and 140 μmol m^–2^ s^–1^; whenever necessary, supplementary illumination was provided with SON-T (600 W, 400 V, Philips Master Greenpower CG, Philips, Eindhoven, Netherlands) high pressure sodium-vapor lamps. To this end the photosynthetic photon flux density was routinely controlled with a quantum sensor (LI-COR, Lincoln, NE, United States). The average relative humidity varied between 74 and 84%; the CO_2_ concentration was 600–900 ppm. The soil in the 1 m wide flower beds was well drained sandy loam with a pH of 6.5 and an EC of 1–1.5. The plants were watered through drip irrigation (8–9 l water m^–2^ day^–1^) and weekly fertigated (20N: 20P: 10K).

#### Flower Type

Flower types were distinct based on the sizes of ray floret and disk floret zones and number and morphology of single florets compared to the original ‘Arjuna’ flower. The ray floret color (FC) was defined with an RHS Large Color Chart. Besides the presence of red spots on ray florets, the formation of completely red ray florets or the presence of a greenish/yellowish disk floret zone was recorded.

In case multiple clearly distinct flower phenotypes were present within the group of 20 cuttings derived from a single mother plant, the group was split and every phenotype was further considered as an independent regenerant and screened separately for the other morphological parameters. These regenerants were further denominated as X(Y)_Z with X = the original regenerant name, Y = the year and Z = the phenotype number. When possible, for every phenotype 5 cuttings from the 2 middle rows were selected for parameter measurements.

#### Flower Initiation

Retention time 1 (RT1) is defined as the number of days between the start of the short day treatment and the presence of 1 at least fully hatched flower on 3–4 plants out of a group of 5. RT3 is the number of days required for the same selection to form 3 fully hatched flowers. All other morphological parameters were measured immediately after the assessment of RT3.

#### Flower and Plant Morphology

The FN is the total number of flowers per plant over all stages, from bud until fully hatched. The FS is the diameter of the total inflorescence (sum of ray and disk florets) of the main flower of the plant. The stalk was cut to determine PS, FW, LW and SW. PS is the total length of this cut stalk, from the cutting edge to the top flower. FW was the combined fresh weight of all flowers of this stalk, buds inclusive; LW was the fresh weight of all its leaves; SW was the fresh weight of the remainder of the stalk after all flowers and leaves had been removed.

FS and PS were measured with a caliper and expressed in cm; FW, LW, and SW were measured with a PCB 200-2 precision balance and expressed in g. Altogether, 2 flower initiation related parameters (RT1 and RT3), 4 flower morphology related parameters (FC, FN, FS, and FW) and 3 parameters related to general morphology (PS, LW, and SW) were measured.

Per phenotype × PA combination, RT1, RT3, FN, FS, FW, LW, SW, and PS were considered exclusively when they were based on a complete group of 5 cuttings. For FC, maximum 5 cuttings were qualitatively scored based on availability. FC was therefore defined for every phenotype irrespective of the number of available cuttings.

#### Flow Cytometry

Per plant a single young leaf was sampled for flowcytometric determination of the relative DNA-content. The samples and internal controls were grinded together with a stainless steel bead in 500 μL citrate buffer (0.1 M citric acid monohydrate, 0.5% Tween20) (Otto, 1990) using a Retsch Tissuelyser II (Qiagen) for 90 s at 30 Hz. Samples were filtered and 750 μL phosphate buffer (0.4 M Na_2_HPO_4_.12H_2_O, 0.1% polyvinylpyrrolidone) with 2 mg L^–1^ 4′,6-diamidino-2-phenylindole (Otto, 1990) was added. The DNA content of the stained nuclei was determined with a Partec Cyflow Space (Sysmex, Münster, Germany) equipped with a 365 nm UV led lamp. Histograms were analyzed using FloMax software (Quantum Analysis, Münster, Germany). The DNA content of the regenerants relative to the DNA content of a hexaploid control plant was determined by calculating the ratio of the DNA content peak position of regenerants to the DNA content peak position of the internal control, according to the formula.

(1) $$\mathrm{Relative}\,\mathrm{DNA}\,\mathrm{content}=\frac{\begin{array}{c} \text{Ratio}(\mathrm{peak}\mathrm{position}\mathrm{regenerant}:\\ \mathrm{peak}\mathrm{position}\mathrm{internal}\mathrm{standard}) \end{array}}{\begin{array}{c} \mathrm{Ratio}(\mathrm{peak}\mathrm{position}\mathrm{control}\mathrm{plant}:\\ \mathrm{peak}\mathrm{position}\mathrm{internal}\mathrm{standard}) \end{array}}$$

In experiment 1, *Phaseolus vulgaris* was used as an internal control; in experiment 2, *P. vulgaris*, *Pisum sativum* or *Zea mays* were used as internal standards. In both experiments, relative DNA-content analysis was based on 1 randomly selected cutting; we considered the mean value of both screenings.

#### Data Processing and Statistical Analysis

All data processing was performed in Statistica 13.1. First, a Shapiro–Wilk’s *W* test was run to evaluate the normality of data distribution within each regenerant × parameter × year combination, for parameters FN, FS, FW, LW, SW, and PS (*n* = 5). In case of normality, regenerants were pairwise compared to V-C with an independent samples *T*-test (*P* < 0.05) for every parameter × year combination; otherwise they were compared to V-C using the Mann–Whitley *U*-test (*P* < 0.05). In case Levene’s test was significant (*P* < 0.05), *T*-tests with separate variance estimates were executed. In addition, in 2018 all regenerants were pairwise compared to T-C.

For both PAs, the differences within the available pool of regenerants were visualized in density plots for RT3, FN, FS, FW, LW, SW, and PS drawn in displayr. To this end, averages were calculated based on the number of available cuttings. All data based on a cutting number ≥1 and ≤5 were considered. Also, a Pearson correlation matrix for these parameters was compiled for both years.

## Results

### Experiment 1

Protoplasts of three cultivars were successfully regenerated: ‘Arjuna’ (18 regenerants, AR1 – AR18), ‘Golden Surfer’ (9 regenerants, GSR1 – GSR9), and ‘Le Bonheur’ (5 regenerants, LBR1 – LBR5) (Table 1). All regenerants were regenerated from different protoplasts and are as such independent regeneration events. Protoplasts of the other 12 cultivars did not regenerate. The protoplast source and culture method differed between the 3 regenerative cultivars. For ‘Arjuna’ regenerative protoplasts were isolated from both leaf and callus cells and grown in liquid medium. For ‘Golden Surfer’ and ‘Le Bonheur’ only mesophyll cells regenerated after culture in liquid medium (‘Le Bonheur’) or beads (‘Golden Surfer’).

**TABLE 1:** Variation in *Chrysanthemum* × *morifolium* ‘Arjuna’, ‘Golden Surfer’ and ‘Le Bonheur’ protoplast regenerants (experiment 1).

**Regenerant**	**Protoplast source**	**^*Y*^Relative DNA content**	**Morphological differences compared to the original cultivar**
‘Golden Surfer’		100	
GSR1	Leaf^*X*^	90	Compact plant; delayed flowering; disk florets less pronounced
GSR2	Leaf^*X*^	84	Compact plant; delayed flowering; looser flower shape
GSR3	Leaf^*X*^	82	Compact plant; delayed flowering
GSR4	Leaf^*X*^	82	Compact plant; delayed flowering
GSR5	Leaf^*X*^	82	Compact plant; delayed flowering
GSR6	Leaf^*X*^	84	Compact plant; delayed flowering
GSR7	Leaf^*X*^	90	Compact plant; delayed flowering
GSR8	Leaf^*X*^	84	Compact plant; delayed flowering
GSR9	Leaf^*X*^	91	Compact plant; delayed flowering
‘Le Bonheur’		100	
LBR1	Leaf	100	None
LBR2	Leaf	100	None
LBR3	Leaf	90	Advanced flowering
LBR4	Leaf	100	None
LBR5	Leaf	100	Compact growth; deformed leaves and flowers
‘Arjuna’		100	
AR1	Callus	100	None
AR2	Callus	ND	None
AR3	Callus	ND	None
AR4	Callus	100	None
AR5	Leaf	100	Incised leaves
AR6	Leaf	90	None
AR7	Callus	ND	None
AR8	Callus	ND	None
AR9	Callus	180	None
AR10	Callus	100	None
AR11	Callus	ND	None
AR12	Callus	ND	None
AR13	Callus	ND	None
AR14	Callus	92	Incised leaves
AR15	Callus	ND	None
AR16	Callus	ND	None
AR17	Callus	180	None
AR18	Callus	ND	None

*^*X*^Protoplasts embedded in LMPA. ^*Y*^Expressed as % of the DNA content ratio of the original cultivar; ND, not determined.*

**FIGURE 2:**
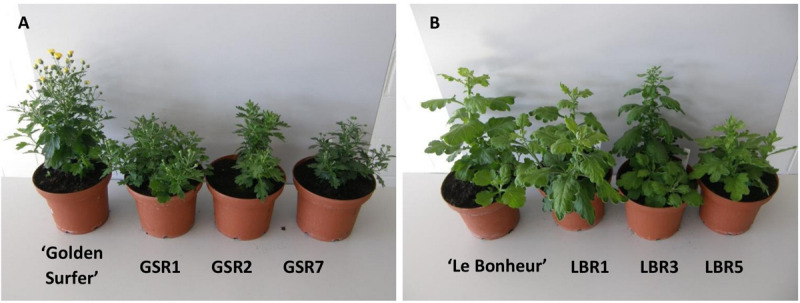
Morphological variation in *Chrysanthemum* × *morifolium* protoplast regenerants: **(A)** ‘Golden Surfer’ regenerants GSR1, GSR2, and GSR7, all with compact growth and delayed flowering; **(B)** ‘Le Bonheur’ regenerants LBR1 (normal phenotype), LBR3 (advanced flower bud formation), and LBR5 (compact growth, deformed leaves).

**FIGURE 3:**
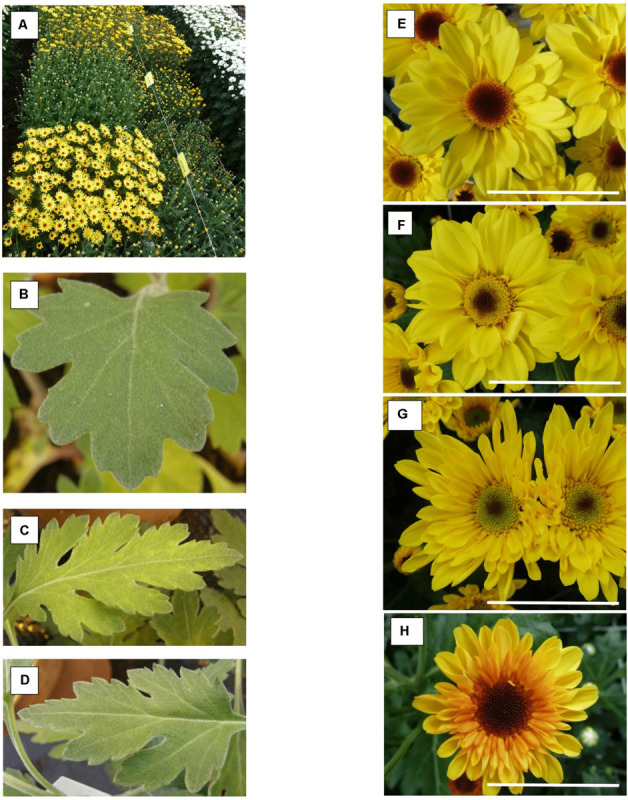
*Chrysanthemum* × *morifolium* ‘Arjuna’ protoplast regenerants, different phenotypes in 2017: **(A)** overview of phenotypic greenhouse trials; **(B)** V-C leaves; **(C)** AR5 leaves; **(D)** AR14 leaves; **(E)** V-C flower; **(F)** yellowish disk florets (RS25); **(G)** greenish disk florets and tubular-type flower (RS37); **(H)** reddish ray florets [RS48(2017)_2]. Scale bar = 5 cm.

Compared to the control all ‘Golden Surfer’ regenerants had a more compact plant habit (Figure 2A) and flowering was delayed. The flower morphology of GSR1 and GSR2 slightly differed from the original flower. In all GSR plants, the DNA content was lower than in the original cultivar and varied between 82% (GSR3, GSR4, GSR5) and 91% (GSR9) (Table 1). Only 1 out of 5 ‘Le Bonheur’ regenerants had an altered DNA content compared to the control. This regenerant, LBR3, flowered earlier than the original cultivar (Figure 2B). Regenerant LBR5 was more compact and formed abnormal leaves and flowers. Most regenerants were obtained from ‘Arjuna’ protoplasts (Figure 3A). In 4 out of 8 regenerants tested, the DNA content had changed. AR9 and AR17 had an increased nuclear mass of 1.8 × ‘Arjuna’; AR6 and AR14 had a smaller nuclear DNA content of, respectively, 90 and 92% of the control. AR5 and AR14 *in vivo* leaves had striking incisions that were lacking in control leaves (Figures 3B–D and Table 1). For the 2nd experiment we selected a regeneration system based on culture of ‘Arjuna’ leaf protoplasts in liquid medium.

### Experiment 2

#### Flower Type

For most regenerants, flower type and color were similar in both test years (Table 2). However, “daisy-types,” “tubular-types,” and “spider-types” were distinct from the regular, “anemone-type” ‘Arjuna’ flower (Table 2). “Daisy-type” flowers have a relatively small ray floret zone, multiple rounds of densely implanted disk florets and a relatively large disk floret zone. “Tubular-type” flowers have all ray florets turned into tubes; the florets, however, still form a consecutive circle. In case all ray florets are tubular but their number is too low to form a consecutive circle, the flowers are “spider-types.” In 2017, 3 out of 54 regenerants (RS2, RS14, and RS48) showed different flower phenotypes within the 20 cuttings (Table 2 and Figures 3E–H). The different types were further considered as separate regenerants, referred to as RS2(2017)_1, RS2(2017)_2, RS14(2017)_1, RS14(2017)_2, RS48(2017)_1, RS48(2017)_2, and RS48(2017)_3. This resulted in a total of 58 regenerants, either with a stable phenotype or a phenotype uniquely observed in 2017. Of these 37 (63.8%) were ‘Arjuna’ (“anemone”)-like, 6 (10.3%) were “spider-types,” 11 (19%) were “tubular-types,” and 4 (6.9%) were “daisy-types.” Moreover, 6 (10.3%) regenerants had ray florets that were partly reddish and 8 (13.8%) regenerants had a greenish or yellowish disk floret zone. Overall, 50% of the regenerants could be distinct from the original ‘Arjuna’ based on flower type and color.

**TABLE 2:** *Chrysanthemum* × *morifolium* ‘Arjuna’ protoplast regenerant phenotypes for extended morphological parameter assessment (experiments 1 and 2) based on superficial flower morphology screening in 2017 and 2018.

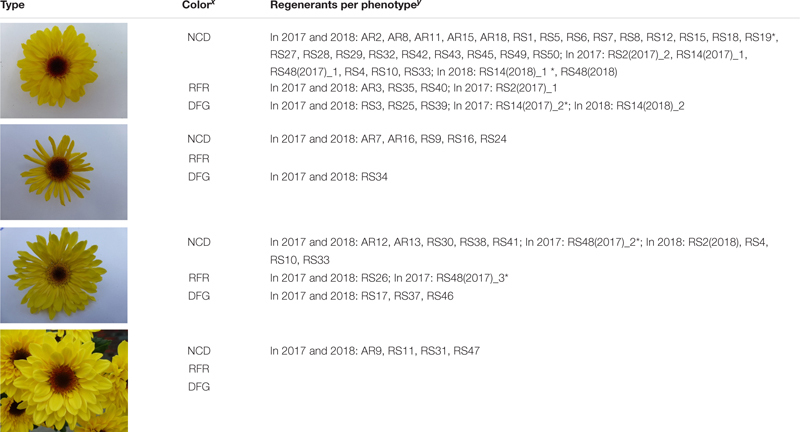

^*x*^NCD: no color deviation (‘Arjuna’-type); RFR: ray florets partly red; DFG: disk florets partly yellow/green. ^*y*^Regenerants not used for PA due to a cutting number <5 are marked with *.

In 2018, 4 regenerants [RS2(2018), RS4, RS10, and RS33] had tubular-type flowers, whereas in 2017 only ‘Arjuna’-type flowers had been observed. RS48(2018) flowers were all identical to the main type observed in 2017 [RS48(2017)_1, the ‘Arjuna’-type]. Only for RS14, 2 different phenotypes were observed, similar to the observations in 2017: RS14(2018)_1, a phenotype with normal disk florets (4 cuttings out of 20) and RS14(2018)_2, a phenotype with partly greenish disk florets (16 cuttings out of 20). Altogether this came down to 55 regenerants; 32 (58.2%) Arjuna-like, 6 (10.9%) “spider-types,” 13 (23.6%) “tubular-types” and 4 (7.3%) “daisy-types”; 23 were visually different from the original ‘Arjuna’ phenotype, partly due to reddish ray florets (4 regenerants, 7.3%) or greenish/yellowish disk florets (8 regenerants, 14.5%).

The main ray floret color of control plants V-C and T-C was always 6A according to the RHS color chart. This brilliant yellow was the most common ray floret color over all regenerant × year combinations. There were some variations in intensity, and other color codes such as 5A, 5B, 6B, 6C, 7A, 7B, 9A, and 9B were perceived on occasion. However, the main ray floret color type was only concisely affected as all recorded colors belong to UPOV color group 11 (yellow).

#### Flower Initiation

**FIGURE 4:**
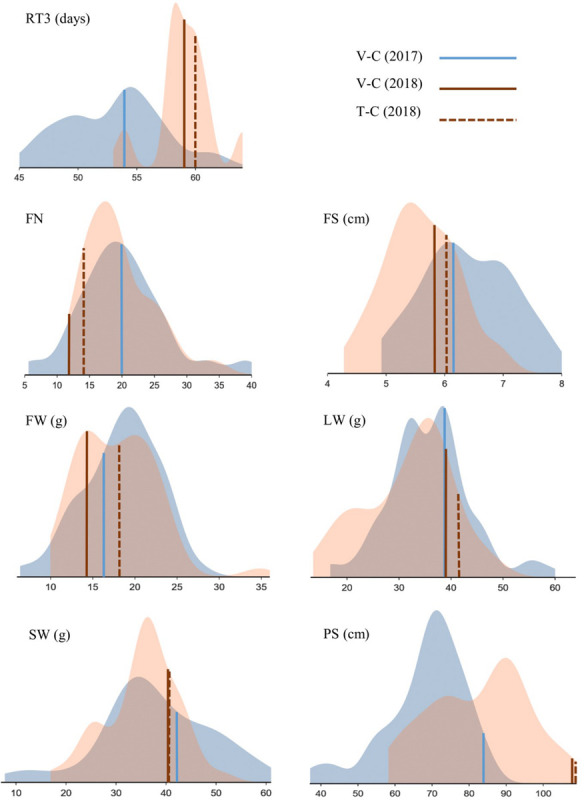
Phenotypic screening of *Chrysanthemum* × *morifolium* ‘Arjuna’ protoplast regenerants: density plots for parameters RT3 (retention time 3), FN (flower number), FS (flower size), FW (flower weight), LW (leaf weight), SW (stalk weight), and PS (plant size) independently assessed in August 2017 (blue) and May 2018 (brown). Controls are indicated.

The retention times RT1 and RT3 were recorded for 37 regenerants in 2017 and 2018 (Supplementary Table 1). In 19 cases (51.3%) all measured RTs showed a reduced or identical flowering time compared to the control V-C (2017) or both V-C and T-C (2018); in 10 cases (27%) the effect was negative (extended or identical flowering time) and in 8 cases (21.6%) flowering time was not consistently reduced or extended. Flowering occurred earlier than in V-C although the effects of tissue culture were minimal based on comparison of V-C and T-C. In general, the effects were most pronounced with regard to RT3 in 2017: this parameter revealed a reduction of the time span required for early flowering of at least a week in 7 regenerants (13%). An RT3 reduction of less than 7 but more than 3 days was furthermore recorded for 11 regenerants (20.4%). On the other hand, flowering time was notably prolonged in 4 regenerants (7.4%); for each of them, RT3 was at least 7 days longer than in V-C; in the case of RS34, RT1 was even prolonged for 10 days. RT3 had notably increased in 2018 in comparison to 2017 (Figure 4).

#### Flower and Plant Morphology

FN, FS, FW, LW, SW, and PS data are summarized in Table 3, presenting the minimal and maximal values, controls and all significantly different regenerants per particular parameter × year combination. The variation per parameter is visualized in Figure 4, and all individual data per regenerant are presented in Supplementary Table 2. Significant phenotypic consequences of protoplast regeneration were observed in cuttings of all regenerants, except for RS(2017)_1 and RS15 in 2017. However, the *in vitro* control T-C cuttings were not statistically different from the *in vivo* control V-C cuttings for any of the measured parameters, indicating that *in vitro* culture as such did not generate somaclonal variation. Overall, 127 out of 304 (41.8%) and 120 out of 297 (40.4%) regenerant parameters significantly deviated from V-C parameters in 2017 and 2018, respectively. In AR plants derived from callus protoplasts and RS plants regenerated from mesophyll protoplasts, respectively, 48/129 (37.2%) and 199/472 (42.2%) parameters significantly differed from V-C parameters.

**TABLE 3:** Morphological parameter assessment of *Chrysanthemum* × *morifolium* ‘Arjuna’ protoplast regenerants after 1 and 2 vegetative multiplication cycles, respectively, assessed in 2017 and 2018 (*n* = 5).

**Parameter (year)**	**Min**	**Max**	**V_C**	**T_C**	**Significantly different regenerant^*x*^**
**FN(2017)**	5.6	39.2	20		AR7, AR9, AR18, RS1, RS3, RS4, RS9, RS12, RS26, RS30, RS34, RS38, RS49
**FN(2018)**	11.8	34.8	11.8	14.2	AR8, AR12*, AR13*, AR16*, RS1*, RS3*, RS5*, RS7*, RS12*, RS14(2018)_2*, RS15*, RS24*, RS26*, RS28*, RS31*, RS32*, RS35*, RS37*, RS40*, RS43, RS49, RS50*
**FS(2017)**	4.92	7.8	6.16		AR7, AR8, AR11, AR12, AR13, RS1, RS2(2017)_2, RS5, RS10, RS11, RS14(2017)_1, RS16, RS17, RS24, RS25, RS27, RS32, RS33, RS35, RS37, RS40, RS43, RS50
**FS(2018)**	4.28	6.92	5.82	6.04	AR2*, AR3*, AR7*, AR18*, RS3*, RS11*, RS16, RS17*, RS27*, RS29, RS30*, RS31*, RS32*, RS33, RS39*, RS40*, RS42*, RS43*, RS47*, RS49*, RS50*
**FW(2017)**	6.38	27.64	16.2		AR7, AR18, RS3, RS5, RS9, RS17, RS18, RS26, RS29, RS33, RS34, RS40, RS42, RS43
**FW(2018)**	9.98	34.78	14.26	18.1	AR12*, AR13*, RS3, RS5*, RS7, RS24, RS26, RS33, RS35, RS37, RS43*, RS50
**LW(2017)**	16.78	59.6	38.8		AR2, AR3, AR7, AR9, AR18, RS1, RS6, RS10, RS11, RS12, RS24, RS26, RS28, RS29, RS33, RS34, RS37, RS38, RS43, RS46
**LW(2018)**	13.48	49.54	38.84	41.56	AR2*, RS4*, RS5*, RS11*, RS12*, RS14(2018)_2*, RS16*, RS17*, RS25*, RS28*, RS29*, RS30*, RS32*
**SW(2017)**	7.76	57.6	42.2		AR7, AR16, AR18, RS3, RS4, RS6, RS9, RS11, RS16, RS26, RS34, RS43, RS49
**SW(2018)**	16.92	51.5	40.34	40.5	AR7*, RS10*, RS30*
**PS(2017)**	37.1	86.8	84		AR2, AR3, AR7, AR8, AR11, AR13, AR16, AR18, RS1, RS2(2017)_2, RS3, RS4, RS5, RS6, RS7, RS8, RS9, RS10, RS11, RS12, RS17, RS18, RS24, RS25, RS27, RS28, RS29, RS30, RS31, RS32, RS33, RS34, RS37, RS38, RS39, RS40, RS41, RS43, RS45, RS46, RS47, RS48(2017)_1, RS49
**PS(2018)**	58.22	108.28	107.68	108.28	AR2*, AR7*, AR8*, AR9*, AR11*, AR12*, AR13*, AR15*, AR16*, AR18*, RS1*, RS2(2018)*, RS3*, RS4*, RS5*, RS7*, RS10, RS11*, RS12*, RS14(2018)_2*, RS15*, RS16*, RS17*, RS18*, RS24*, RS25*, RS26*, RS27*, RS28*, RS29*, RS30*, RS31*, RS32*, RS33*, RS35*, RS37*, RS38*, RS39*, RS40*, RS41*, RS42*, RS43*, RS45*, RS46*, RS47*, RS48(2018)*, RS49*, RS50*

*^*x*^Regenerants significantly different from in vitro control T_C are indicated with ^∗^. FN, flower number; FS, flower size; FW, flower weight; LW, leaf weight; SW, stalk weight; PS, plant size. Per parameter × year combination, regenerants significantly different (Tukey, P < 0.05) from an in vivo control V_C are listed.*

For FN, in 2017 13 out of 52 regenerants were significantly different from V-C. Unlike in 2017, in 2018 the V-C cuttings produced the lowest FN, and almost half of the regenerants (22/49) had more flowers than V-C; of these 22, 19 (38.8%) also differed from the *in vitro* control T-C. The variation between regenerants was substantial, particularly in 2017 (Table 3). With regard to FS, the ratio of significantly different regenerants was 23/44 (52.3%) and 21/46 (45.7%) for 2017 and 2018, respectively. Eighteen regenerants (39.1%) were different from both V-C and T-C with respect to FS in 2018. As shown in Figure 4, flowers were larger in 2017 than in 2018, but variation between regenerants was fairly high in both years. The last flower related parameter, FW, was significantly different from V-C for 14/52 (26.9%) regenerants in 2017 and for 12/49 (24.5%) regenerants in 2018. Of the latter group 4 regenerants (8.2%) also significantly diverge from T-C. There was no notable shift in FW in 2018 in comparison to 2017. The FW of most regenerants was higher than that of the V-C controls in both years (Figure 4). The LW recorded in 2017 significantly deviated from the LW of V-C for 20 out of 52 regenerants (38.5%); in 2018 this was the case for 13 out of 49 regenerants (26.5%), with all of them also differing from T-C (Table 3). On average LW values were not higher or lower in 2018 than in 2017, although again a notable variation was observed within the regenerant assortment. SW was significantly different between V-C and 13/52 regenerants (25%) in 2017 and 3/49 (6.1%) regenerants in 2018, the latter also being significantly dissimilar with T-C. Although the SWs for all controls were similar, also for this parameter an extended variation is visualized in Figure 4. PS was the parameter for which most significant differences were recorded in both years (Supplementary Table 2). Control plants V-C and T-C were significantly taller than the majority of the regenerants (Figure 4). Plants were on average taller in 2018 than in 2017. In 2017, 43 out of 52 regenerants (82.7%) were significantly smaller than V-C and in 2018 the PS of 48 out of 49 regenerants (98%) was significantly reduced compared to V-C, among which 47 (95.9%) also differed from T-C.

All measured regenerants significantly differ from the original cultivar; yet, some stand out more than others. A list comprising the most atypical regenerants was compiled. In 2017, AR7 significantly differed from V-C for all parameters measured, and AR18, RS3, RS34, and RS43 for all but one. Moreover, for RS3 and RS43 also in 2018 4 out of 6 statistically processed parameters significantly deviated from V-C. Also RS6, RS9 and RS14(2018)_2 were notably different from the control albeit their parameters could only be measured once.

#### Parameter Correlations

**FIGURE 5:**
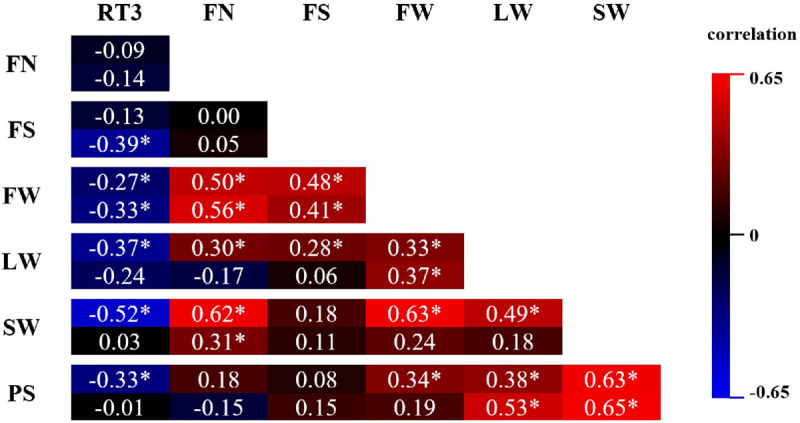
Pearson correlation matrix of phenotypic parameters FN (flower number), FS (flower size), FW (flower weight), LW (leaf weight), SW (stalk weight), and PS (plant size) assessed on *Chrysanthemum* × *morifolium* ‘Arjuna’ protoplast regenerants in 2 independent trials in 2017 (upper value) and 2018 (lower value). Significant correlations (*P* < 0.05) are labeled with *.

In Figure 5, pairwise Pearson correlations between RT3 and all statistically processed parameters are shown for both years. All significant correlations of RT3 with any of the other parameters is negative. The strongest correlation of RT3 (−0.52) is with SW in 2017, although in 2018 the correlation was very weak and insignificant. The absolute value of all other RT3 correlations is lower than 0.4. On the other hand, all significant correlations between FN, FS, FW, LW, SW, and PS are positive. Correlations ≥0.5, yet <0.7 were recorded between FN and FW (both years), FN and SW (2017), FW and SW (2017), LW and PS (2018), and SW and PS (both years). Correlations were significant for both years in 7 parameter combinations; in 2 out of these 7 combinations there was a notable difference between correlations for both years (FN-SW and LW-PS).

#### Flow Cytometry

The relative DNA amount of the 48 stable phenotypes from Table 2 was assessed. Seven phenotypes had a genome size aberration of at least 5% compared to V-C: RS40 (88.4%), AR18 (92.5%), RS6 (93.6%), RS45 (94.1%), RS9 (176%), AR7 (180%), and RS34 (181%).

## Discussion

The main objective of this study was to evaluate the stability of *Chrysanthemum* × *morifolium* protoplast regenerants and to quantify commercially relevant morphological changes induced by somaclonal variation (Larkin and Scowcroft, 1981) linked to protoplast regeneration.

A first experiment was set up to test the protoplast regeneration potential of a series of pot and cut chrysanthemum cultivars in order to select a particular protocol for the production of somaclones and the subsequent assessment of their morphology. Deformed leaves and flowers, delayed or advanced flowering, compact growth and altered genome size were observed, depending on the genotype. This is consistent with publications of Malaure et al. (1991) and Zalewska et al. (2007) who previously demonstrated the genotype dependency of somaclonal variation in *Chrysanthemum*. Based on this experiment, four strategies were possible to supply protoplast regenerants: (i) regeneration of ‘Arjuna’ callus protoplasts in liquid medium, (ii) regeneration of ‘Arjuna’ leaf protoplasts in liquid medium, (iii) regeneration of ‘Golden Surfer’ leaf protoplasts through culture in LMPA beads and (iv) regeneration of ‘Le Bonheur’ leaf protoplasts in liquid medium. Mesophyll cells are better starting material than callus cells because the initiation of callus cells requires an extra *in vitro* step that is likely cultivar dependent and may have an effect on protoplast regeneration efficiency later on. Moreover, protoplast isolation of leaf cells requires lower enzyme concentrations than from callus. The application of LMPA solidified beads is labor intensive compared to protoplast culture in liquid medium. ‘Arjuna’ is the only cultivar from which protoplasts were regenerated in 2 independent experiments. For these reasons, we selected a regeneration system based on culture of ‘Arjuna’ leaf protoplasts in liquid medium.

Following, we regenerated ‘Arjuna’ protoplasts and measured an array of relevant morphological parameters in 2 independent screening trials. These trials were performed in different periods of the year (August 2017 vs. May 2018) to test the reproducibility of any variation under different physiological circumstances. For these reasons the trials should not be considered as repeats. The parameters were carefully selected to provide as well a thorough description on general plant morphology and to reflect the underlying plant physiological status. To perform these trials, cuttings were produced from every independent regenerant. Although cuttings produced from the same regenerant were expected to be genetic copies, morphological variation occurs in some cases, as well in 2017 (3 regenerants) as in 2018 (1 regenerant). These variations were disk floret discoloration, ray floret discoloration or changes in ray floret morphology and are an indirect indication of chimerism in the mother plants. We considered these morphological aberrations as separate, independent events and screened them accordingly. The main ray floret color consistently belonged to color group 11 (yellow) and only minor color deviations were recorded.

### All Protoplast Regenerants Are Affected by Protoclonal Variation

For all protoplast regenerants, significant effects on flower and/or plant morphology were quantified. On the other hand, *in vitro* control plants (T-C), grown from shoots multiplied through tissue culture, did not significantly differ from *in vivo* control plants (V-C) for any measured parameter. This indicates that the observed variations are no mere *in vitro* effect but that protoplasting is indispensable to induce this somaclonal formation. Based on the similar phenotypic consequences in AR and RS plants, that were generated from different experiments, we speculate that protoplasting on itself has more effects on regenerant morphology than the exact protoplast source.

The rate of somaclonal variation we observed after protoplast culture is much higher than after regeneration from other *Chrysanthemum* explant types. Khalid et al. (1989) obtained 2.5% somaclonal variation after leaf regeneration and 43% after petal regeneration. Miler and Jedrzejczyk (2018) described somaclonal variation obtained after *Chrysanthemum* ovary culture with regard to either leaf or inflorescence morphology, in 16.4% of the regenerants. The affected parameters in our study were consistent with previously published literature on somaclonal variation in *Chrysanthemum*. Kengkarj et al. (2008) cultured *Chrysanthemum* petal segments and induced somaclonal variation that was mainly inflorescence related. Although the variations were not quantified, their type (altered ray floret shape, number of inflorescences, plant weight, plant height and LW) is conform with our own observations.

The regenerants that differed from the controls were not consistently the same in both years. Possibly, some of the original regenerants were chimeric, resulting in different mother plants and/or cutting batches in 2017 versus 2018. On the other hand, physiological differences between plants screened in August (2017) versus plants screened in May (2018) are likely to result in altered morphologies as well. These differences are illustrated in Figure 4, showing relatively more vegetative plant development in 2018 than in 2017 (taller plants, longer flowering induction time and smaller flowers). It would therefore be incorrect to compare data from both years pairwise; nonetheless, there are pronounced common tendencies. As well in 2017 and in 2018, PS was the parameter that was most affected by protoplast regeneration, whereas SW was relatively the least affected. Also, for all statistically processed parameters an extensive variation is apparent. For neither year, strong correlations between parameters were calculated, indicating their complementarity for phenotypic screening.

### Somaclones Are Mutually Divergent

Protoclonal variation was omnipresent in our regenerant pool; yet, there were no reoccurring phenotypes and the morphology within the regenerant set was very diverse. For instance, as well shortening as extension of flowering time were observed in ‘Arjuna’, consistent with as well our own observations in ‘Golden Surfer’ and ‘Le Bonheur’ and protoplast culture induced somaclonal variation in *Dianthus* (Shiba and Mii, 2005). This suggests that more substantial variation within protoplast regenerants is still possible, even within the ‘Arjuna’ cultivar, and that it is recommended to use an efficient regeneration protocol that enables the regeneration of hundreds of plants to fully exploit the potential of a particular genotype for protoclonal variation.

AR7, AR18, RS3, RS6, RS9, RS14(2018)_2, RS34, and RS43 were the regenerants with the most significantly different parameters compared to V-C. Within this list, AR7, RS9 and RS34 had a spider-type inflorescence and RS3, RS14(2018)_2 and RS34 had greenish disk florets.

Relative DNA ratios were determined for all regenerants. As opposed to Miler and Jedrzejczyk (2018) who did not find any ploidy alterations among regenerants at all, we detected 7 regenerants with 5% or more genome size aberration. A cross-reference of this regenerant list and the abovementioned list of 8 regenerants with most significant phenotypic alterations yields 5 common phenotypes. AR7, RS9 and RS34 have a spider-type inflorescence that is possibly linked to their increased genome size, although also 3 other regenerants with a regular genome size bear this type of inflorescence. AR18 and RS6, that are also morphologically notably distinct from V-C, have a slightly reduced genome. Only 2 regenerants with modified ploidy level, RS40 and RS45, do not belong to the group of most atypical regenerants; conversely, only 3 out of 8 of the most atypical regenerants [RS3, RS14(2018)_2, RS43] have a regular genome size. This points toward a causative effect of genome size modification on protoclonal variation, e.g., through chromosome loss or polyploidization. We speculate that the genome contents measured for RS40 (88.4%), AR18 (92.5%), RS6 (93.6%), and RS45 (94.1%) are not consistent with the loss of an entire genome that we would expect to account for 16.67% nuclear mass loss based on the hexaploidy of the original plant. The loss of a number of chromosomes therefore is more plausible than the loss of an entire chromosome set, but this assumption can only be verified by chromosome counting. The driver behind the increased genome size of RS9 (176%), AR7 (180%) and RS34 (181%) is probably chromosome loss combined with spontaneous duplication rather than with spontaneous protoplast fusion, as the latter phenomenon was never microscopically observed in our fusion experiments (data not shown). This is in accordance with the observation that the genome sizes of RS9, AR7, and RS34 are about double the genome sizes of RS40, AR18, RS6, and RS45.

AR9 exhibited an 80% higher DNA content ratio after the first experiment but a DNA content ratio similar to the one of the control plants after the second experiment. We speculate that this is the consequence of chimerism in the original regenerant. Another possible explanation is that chromosome number alterations are not always stable, resulting in chromosome loss from aneuploid regenerants derived from protoplast regeneration. In both scenarios it would be recommended to pass the somaclones through a number of vegetative cycles to achieve karyotypic stability. As well chimerism and temporary somaclone instability are consistent with the presence of multiple phenotypes among flowering cuttings of regenerants RS2, RS14, and RS48 in one or both years.

After the screening of regenerants from the first experiment all ‘Golden Surfer’ regenerants had a reduced genome size compared to the control, hinting at genotype effects on protoclonal variation in *Chrysanthemum*. This study did not aim to unravel particular genotype effects or molecular backgrounds of the different types of protoclonal variations that were documented. Yet, the high potential of protoplasts as source material for somaclone production is intriguing. One possible effect of protoplast culture is the regeneration of plants with an epigenetically altered expression level of morphology defining genes such as the *CYC2* genes that influence the capitulum type in the Asteraceae family (Chen et al., 2018). In *Cucumis*, dedifferentiation of mesophyll cells into protoplasts is followed by heterochromatin reduction, with the subsequent chromocenter re-establishment depending on the exact protoplast culture method (Ondrej et al., 2009). This heterochromatin decondensation upon dedifferentiation is also observed in *Arabidopsis* (Tessadori et al., 2007). In *Solanum tuberosum*, a model crop for protoplast studies thanks to its high regenerability, Fossi et al. (2019) demonstrated large scale copy number changes, aneuploidy, and segmental deletions and duplications that exclusively occur in protoplast regenerants and not after regular cutting propagation. Cultivated potato is a tetraploid and the authors expect fewer large-scale abnormalities would occur in regenerants from diploids because gross dosage and structural variants will be selected against during regeneration. Yet, *Chrysanthemum* is a hexaploid and therefore the abovementioned variations could occur on an even more pronounced scale than in *S. tuberosum*. Also, persistent instability was indicated in leaves of the same *Solanum* somaclone (Fossi et al., 2019). This could be consistent with the formation of different phenotypes from a single regenerant in our experiments (RS2, RS14, and RS48) and with the unstable genome size of AR9. It is very likely that the multiple genomic changes demonstrated through sequencing in *Solanum* are linked to the phenotypic alterations in protoplast regenerants. In strawberry ‘Chandler’ protoplast regenerants, EST-SSR markers demonstrated genetic changes in all analyzed regenerant lines, but ploidy had remained unaffected. The genetic changes in these regenerants could be linked to the observed fruit yield reduction (Barceló et al., 2019).

### Somaclone Production Through Protoplasting Might Have Economic Potential

Contemporary chrysanthemum breeding is based on conventional crossing and induced mutagenesis. Many of the morphological aberrations described in this manuscript for ‘Arjuna,’ ‘Golden Surfer,’ or ‘Le Bonheur’ are also typically induced after mutation treatments in chrysanthemum, as described in the reviews of Rusli et al. (2018) and Yamaguchi (2018). *Chrysanthemum* is a crop particularly prone to mutation induction, with mutants accounting for almost 40% of the number of officially registered cultivars. Especially with regard to petal colors, irradiation with ion beams, chronic exposure to gamma rays and treatment with EMS have created novelties in the assortment. Apart from altered petal colors, dwarf mutants or mutants with fewer lateral buds, early flowering, changed floret shape or reduced DNA content have been described, but manipulation of the inflorescence color(s) is the main driver for mutagenesis breeding in chrysanthemum.

The mutation rate of irradiated or EMS treated explants is genotype dependent, but, similar to protoplast regeneration, plant material type is a defining parameter with regard to mutation efficiency. When petals, leaves and flower buds are irradiated and subsequently regenerated through a callus phase the mutation rate is higher in petal regenerants (reviewed by Rusli et al., 2018). This is similar to the observations of Khalid et al. (1989) who described petals as the source material that is most efficient to induce somaclonal variation. It might be speculated that either these mutations or somaclonal variations are linked to a tissue specific gene expression profile and that therefore the effect of protoplasting would likely differ depending on the source tissue used for protoplasts, creating opportunities to increase the rate of somaclonal variation. In our study, only leaves and leaf derived callus were used as protoplast source; to our knowledge a protocol to regenerate plants from chrysanthemum petal protoplasts has not yet been described.

Although somaclonal variation is undesirable in commercial micropropagation that aims to produce true-to-type plants, plant breeders may use it as a tool to rapidly obtain phenotypic variability without the need for sophisticated technology. Indeed, the process can be manipulated by genotype, tissue source, explant preparation, media composition or physical environment (Karp, 1995; Krishna et al., 2016). For instance, tissue culture of pot azalea leaf explants increases the frequency of flower color bud sporting (Samyn et al., 2002). Somaclones are not bound to the legal restrictions or containments associated with transgenic plants and are therefore readily available for commercialization. Several publications (Bush et al., 1976; Miyazaki and Tashiro, 1978; Sutter and Langhans, 1981; Khalid et al., 1989; Malaure et al., 1991; Zalewska et al., 2007; Kengkarj et al., 2008; Miler and Zalewska, 2014; Okamura et al., 2015; Miler and Jedrzejczyk, 2018) report somaclonal variation from various *Chrysanthemum* explants and some of these authors have evaluated the commercial potential of the respective somaclones. Our own observations pinpoint the particular innovative potential of protoplasts as source material for protoclonal variation. This could result in more applications than purely the modification of ornamental properties. Grzebelus et al. (2013) identified protoclonal variants with increased *Alternaria* tolerance after exposing carrot protoplasts to the fungus filtrate. Likewise, biotic stress exposure of *Chrysanthemum* protoplasts may provide a tool to enhance tolerance to particular diseases in regenerants via protoclonal variation.

As a drawback, results of somaclonal variation are unpredictable. It is also impossible to introgress particular DNA sequences or genes through somaclonal variation. Furthermore, when using protoplasts as source explants, it should be kept in mind that regeneration is the most common bottleneck in any protoplast related work (Davey et al., 2005; Eeckhaut et al., 2013) and as a result this strategy is confined to a limited number of species, including *Chrysanthemum*. Nonetheless, contemporary tools like next generation sequencing, metabolite profiling and *in situ* hybridization offer prospects to gain insight in the regeneration process and consequently to expand the group of regenerative species. Protoplasts can be excellent targets for genome editing techniques such as CRISPR/Cas9, especially in combination with DNA free technologies as RNP (Woo et al., 2015) but the spontaneous formation of somaclones may result in variation among the regenerants that is not caused by the genome editing; for that reason results on genome editing through protoplasts should be considered with caution.

From a scientific point of view, we can conclude that the *Chrysanthemum* protoplasts in our study were very susceptible to protoclonal variation. Oxidative stress is common in tissue culture and may provoke somaclonal variation (Bednarek and Orlowska, 2019), and the amount of oxidative stress to which protoplasts are exposed is substantially higher than for most other tissues (Smulders and de Klerk, 2011; Eeckhaut et al., 2013). The phenotypical effect varies between cultivars but also within our test cultivar ‘Arjuna,’ possibly reflecting different biochemical mechanisms of protoclonal variation. In the most aberrant protoclones, phenotypic variation often goes hand in hand with a modified 2C level. Several indications for the occurrence of chimeras among the regenerants were observed.

As a practical conclusion, we state that regeneration of protoplasts of *Chrysanthemum* × *morifolium* has potential to introduce novelties in the commercial assortment thanks to the spontaneous formation of somaclones. The extent of this potential will be genotype dependent, but for the test cultivar ‘Arjuna’ statistically different phenotypes with regard to flowering and general morphology, reflecting underlying effects on plant physiology, were assessed. The mutual variation within this series of phenotypes was very notable, as was the difference between both independent screenings.

## Data Availability Statement

The raw data supporting the conclusions of this article will be made available by the authors, without undue reservation.

## Author Contributions

JV, TE, and WV: experimental design. WV, TE, SB, and LL: practical work. TE and LL: data processing. TE: writing of the manuscript. SB, LL, and JVH: critical revision of the manuscript. All authors contributed to the article and approved the submitted version.

## Conflict of Interest

The authors declare that the research was conducted in the absence of any commercial or financial relationships that could be construed as a potential conflict of interest.

## Abbreviations

AR: regenerant produced in experiment 1 (1st series)
FC: ray floret color
FN: flower number
FS: flower size
FW: flower weight
KMV: Kao and Michayluk vitamins
LMPA: low melting point agarose
LW: leaf weight
MSS: Murashige and Skoog salts
PA: parameter assessment
PS: plant size
RS: regenerant produced after experiment 1 (2nd series)
RT1: retention time 1
RT3: retention time 3
SW: stalk weight
T-C: *in vitro* control
V-C: *in vivo* control.
