# Supplementary material for: Somaclonal Variation in Chrysanthemum × morifolium Protoplast Regenerants
Source: Front Plant Sci. 2020 Dec 18;11:607171. doi: 10.3389/fpls.2020.607171 (PMC7775395; doi:10.3389/fpls.2020.607171)
Supplement: Supplementary file 1 [file Data_Sheet_1.docx]

Supplementary Material

# Supplementary Tables

**Supplementary table 1.** RT1 and RT3 for *Chrysanthemum* x *morifolium* ‘Arjuna’ protoplast regenerants after 1 and 2 vegetative multiplication cycles, respectively assessed in 2017 and 2018.

| **regenerant** ^x^ | **RT1 (days)** | | **RT3 (days)** | | **regenerant** | **RT1 (days)** | | **RT3 (days)** | |
| --- | --- | --- | --- | --- | --- | --- | --- | --- | --- |
|  | **2017** | **2018** | **2017** | **2018** |  | **2017** | **2018** | **2017** | **2018** |
| **V-C** | 51 | 56 | 54 | 59 | **RS16** | 50 | 55 | 54 | 58 |
| **T-C** | NI | 58 | NI | 60 | **RS17** | 50 | 56 | 54 | 58 |
| **AR2** | 51 | 58 | 54 | 61 | **RS18** | 47 | 54 | 50 | 58 |
| **AR3** | 55 | MD | 57 | 60 | **RS24** | 47 | 54 | 50 | 58 |
| **AR7** | 52 | MD | 54 | 60 | **RS25** | 48 | MD | 50 | 58 |
| **AR8** | 49 | 55 | 51 | 59 | **RS26** | 50 | 58 | 54 | 60 |
| **AR9** | 50 | 55 | 51 | 59 | **RS27** | 53 | 58 | 55 | 61 |
| **AR11** | 47 | 52 | 49 | 54 | **RS28** | 48 | MD | 51 | 58 |
| **AR12** | 49 | MD | 54 | 58 | **RS29** | 50 | MD | 54 | 58 |
| **AR13** | 46 | MD | 48 | 59 | **RS30** | 53 | MD | 55 | 64 |
| **AR15** | 54 | MD | 57 | 64 | **RS31** | 45 | 53 | 47 | 54 |
| **AR16** | 41 | 52 | 45 | 53 | **RS32** | 51 | MD | 54 | 58 |
| **AR18** | 51 | 58 | 54 | 60 | **RS33** | 46 | 53 | 47 | 58 |
| **RS1** | 50 | 56 | 54 | 58 | **RS34** | 61 | ND | 62 | ND |
| **RS2*** | 50 | 60 | 55 | 61 | **RS35** | 46 | 54 | 48 | 59 |
|  | 52 |  | 57 |  | **RS37** | 51 | 57 | 54 | 61 |
| **RS3** | 57 | 58 | 61 | 60 | **RS38** | 50 | 56 | 54 | 59 |
| **RS4** | 50 | 56 | 57 | 58 | **RS39** | 50 | 59 | 54 | 60 |
| **RS5** | 46 | 54 | 47 | 58 | **RS40** | 54 | 61 | 57 | 64 |
| **RS6** | 54 | ND | 57 | ND | **RS41** | 50 | 58 | 51 | 60 |
| **RS7** | 50 | 58 | 57 | 61 | **RS42** | 59 | MD | 61 | 64 |
| **RS8** | 53 | ND | 61 | ND | **RS43** | 45 | 53 | 47 | 54 |
| **RS9** | 54 | ND | 57 | ND | **RS45** | 46 | 54 | 50 | 59 |
| **RS10** | 45 | 52 | 47 | 54 | **RS46** | 46 | 54 | 49 | 59 |
| **RS11** | 50 | 58 | 54 | 60 | **RS47** | 53 | 57 | 55 | 60 |
| **RS12** | 53 | 58 | 55 | 61 | **RS48***** | 54 | 57 | 57 | 60 |
| **RS14**** | 45 | 55 | 47 | 58 | **RS49** | 48 | 58 | 50 | 59 |
| **RS15** | 48 | 55 | 50 | 58 | **RS50** | 49 | 58 | 50 | 59 |

^x^ *results for RS2(2017)_1, RS2(2017)_2 and RS2(2018); ** results for RS14(2017)_1 and RS14(2018)_2; ***results for RS48(2017)_1 and RS48(2018).

RT1: retention time 1; RT3: retention time 3; V-C: in vivo control; T-C: in vitro control; ND: not determined (less than 5 cuttings available); NI: not included; MD: missing data (RT1 had already past at the moment of observation).

**Supplementary table 2.** Variation of morphological parameters (FN, FS, FW, LW, SW and PS) for *Chrysanthemum* x *morifolium* ‘Arjuna’ protoplast regenerants after 1 and 2 vegetative multiplication cycles, respectively assessed in 2017 and 2018 (n=5). All individual regenerants were compared pairwise to V-C (2017) and V-C and T-C (2018) for every parameter through an independent samples T-test (P < 0.05) in case data were normally distributed, and through a Mann-Whitney U test (P < 0.05) in case data were not normally distributed.

| **Regenerant** | **FN** | | **FS (cm)** | | **FW (g)** | | **LW (g)** | | **SW (g)** | | **PS (cm)** | |
| --- | --- | --- | --- | --- | --- | --- | --- | --- | --- | --- | --- | --- |
|  | **2017** ^w^ | **2018** ^w,x,y^ | **2017** ^w^ | **2018** ^w,x^ | **2017** ^w^ | **2018** ^w,x^ | **2017** ^w^ | **2018** ^w,x^ | **2017** ^w^ | **2018** ^w,x^ | **2017** ^w,z^ | **2018** ^w,x^ |
| **V-C** | 20.0 | 11.8 | 6.16 | 5.82 | 16.20 | 14.26 | 38.80 | 38.84 | 42.20 | 40.34 | 84.00 | 107.68 |
| **T-C** | NI | 14.2 | NI | 6.04 | NI | 18.10 | NI | 41.56 | NI | 40.50 | NI | 108.28 |
| **AR2** | 27.0 | 17.8 | 4.92 | 4.92*+ | 17.40 | 13.36 | 25.00* | 24.08*+ | 32.00 | 26.38+ | 69.60* | 86.40*+ |
| **AR3** | 26.6 | 17.0 | ND | 4.46*+ | 18.60 | 13.38 | 30.80* | 30.46 | 41.00 | 43.34 | 79.80* | 107.42 |
| **AR7** | 11.2* | 15.2 | 5.42* | 4.28*+ | 10.96* | 14.34 | 31.00* | 29.04+ | 21.20* | 23.18*+ | 56.00* | 62.54*+ |
| **AR8** | 26.6 | 20.0* | 5.44* | 5.28 | 19.22 | 18.22 | 42.20 | 40.08 | 51.80 | 37.43 | 78.80* | 100.38*+ |
| **AR9** | 14.8* | 13.2 | 5.86 | 6.26 | 16.60 | 19.34 | 45.60* | 40.44 | 43.40 | 36.42 | 84.40 | 104.48*+ |
| **AR11** | ND | 15.0 | 7.00* | 6.10 | 19.28 | 15.24 | 38.60 | 49.54+ | 44.80 | 38.41 | 80.40* | 99.96*+ |
| **AR12** | 18.0 | 25.0*+ | 6.98* | 5.38 | 21.78 | 23.35*+ | 32.20 | 42.16 | 38.40 | 35.19 | 78.20 | 93.18*+ |
| **AR13** | 24.6 | 25.0*+ | 6.80* | 5.54 | 20.00 | 23.12*+ | 35.00 | 39.47 | 50.80 | 34.17 | 73.20* | 96.22*+ |
| **AR15** | 23.2 | 15.4 | ND | 5.14+ | 17.20 | 13.30+ | 37.20 | 31.36+ | 36.00 | 41.50 | 77.40 | 104.20*+ |
| **AR16** | 21.6 | 24.2*+ | 6.50 | 5.42+ | 15.86 | 19.71 | 34.60 | 44.65 | 28.60* | 30.37 | 67.40* | 84.82*+ |
| **AR18** | 14.8* | 17.8 | 6.24 | 5.24*+ | 12.80* | 14.30 | 32.40* | 35.90 | 29.00* | 36.64 | 73.40* | 89.92*+ |
| **RS1** | 13.4* | 19.2*+ | 6.78* | 6.24 | 15.00 | 17.08 | 45.60* | 29.32+ | 34.80 | 39.54 | 76.60* | 91.22*+ |
| **RS2(2017)_1** | 20.0 | NI | ND | NI | 20.20 | NI | 26.60 | NI | 43.80 | NI | 79.60 | NI |
| **RS2(2017)_2** | 22.8 | NI | 7.00* | NI | 17.80 | NI | 39.20 | NI | 36.40 | NI | 69.80* | NI |
| **RS2(2018)** | NI | 13.6 | NI | 5.58 | NI | 11.60+ | NI | 36.58 | NI | 38.62 | NI | 87.98*+ |
| **RS3** | 27.4* | 33.0*+ | ND | 5.38*+ | 12.40* | 19.86* | 46.20 | 35.76 | 30.40* | 35.80 | 53.00* | 73.06*+ |
| **RS4** | 15.0* | 18.0+ | ND | ND | 20.40 | 13.72+ | 37.60 | 21.68*+ | 31.40* | 33.92 | 65.60* | 68.42*+ |
| **RS5** | 17.0 | 27.6*+ | 6.82* | 6.30 | 23.20* | 25.20*+ | 41.60 | 27.32*+ | 44.00 | 34.78 | 71.20* | 87.16*+ |
| **RS6** | 17.0 | ND | ND | ND | 12.68 | ND | 25.02* | ND | 14.04* | ND | 44.80* | ND |
| **RS7** | 20.2 | 21.8*+ | 6.88 | 5.68 | 23.12 | 21.82* | 40.40 | 35.94 | 34.60 | 32.42 | 68.30* | 76.62*+ |
| **RS8** | 33.4 | ND | ND | ND | 27.64 | ND | 42.80 | ND | 33.20 | ND | 62.40* | ND |
| **RS9** | 6.8* | ND | 6.52 | ND | 9.20* | ND | 31.80 | ND | 12.44* | ND | 37.10* | ND |
| **RS10** | 19.2 | 14.6 | 6.92* | 5.48+ | 19.16 | 16.66 | 48.86* | 38.72 | 35.70 | 20.32*+ | 60.20* | 61.78* |
| **RS11** | 21.4 | 14.4 | 5.14* | 4.68*+ | 14.00 | 11.82+ | 26.20* | 18.66*+ | 28.60* | 26.64 | 65.80* | 68.84*+ |
| **RS12** | 39.2* | 25.4*+ | ND | 4.64 | 19.20 | 14.56 | 21.80* | 22.46*+ | 51.00 | 36.30 | 60.60* | 69.76*+ |
| **RS14(2017)_1** | ND | NI | 7.10* | NI | ND | NI | ND | NI | ND | NI | ND | NI |
| **RS14(2017)_2** | ND | NI | ND | NI | ND | NI | ND | NI | ND | NI | ND | NI |
| **RS14(2018)_1** | NI | ND | NI | ND | NI | ND | NI | ND | NI | ND | NI | ND |
| **RS14(2018)_2** | NI | 34.8*+ | NI | ND | NI | 21.06 | NI | 13.48*+ | NI | 43.54 | NI | 68.14*+ |
| **RS15** | 21.0 | 25.4*+ | 6.31 | 5.96 | 17.60 | 18.62 | 41.60 | 28.58+ | 57.60 | 47.14 | 83.40 | 94.58*+ |
| **RS16** | 12.4 | 16.4 | 7.72* | 6.35* | 17.00 | 11.66+ | 39.40 | 22.64*+ | 28.60* | 32.80 | 73.60 | 73.68*+ |
| **RS17** | 16.0 | 16.4 | 7.60* | 6.89*+ | 23.20* | 18.30 | 34.60 | 27.06*+ | 39.60 | 35.70 | 86.80* | 99.58*+ |
| **RS18** | 21.0 | 18.8 | 5.82 | 5.26+ | 24.40* | 20.61 | 31.60 | 38.32 | 52.80 | 35.74 | 80.40* | 90.86*+ |
| **RS19** | ND | ND | ND | ND | ND | ND | ND | ND | ND | ND | ND | ND |
| **RS24** | 21.6 | 18.8*+ | 7.06* | 5.92 | 21.18 | 23.43* | 33.00* | 37.34 | 45.80 | 31.84 | 72.00* | 80.26*+ |
| **RS25** | 19.0 | 17.2 | 5.52* | 5.37+ | 15.88 | 11.98+ | 43.60 | 19.06*+ | 33.00 | 24.78+ | 76.20* | 76.76*+ |
| **RS26** | 12.0* | 24.0*+ | 6.00 | 5.56+ | 11.60* | 20.96* | 32.60* | 33.72+ | 22.80* | 37.42 | 78.80 | 90.82*+ |
| **RS27** | 22.6 | 14.8 | 4.96* | 5.10*+ | 13.80 | 13.94+ | 47.20 | 44.42 | 33.60 | 31.78 | 63.40* | 75.98*+ |
| **RS28** | 17.4 | 28.0*+ | 5.86 | ND | 22.46 | 21.20 | 31.60* | 15.56*+ | 46.60 | 51.50 | 72.00* | 73.92*+ |
| **RS29** | 17.6 | 17.6 | 6.56 | 6.37* | 21.20* | 14.40 | 29.60* | 18.42*+ | 33.20 | 25.58+ | 57.60* | 58.22*+ |
| **RS30** | 32.6* | 12.6 | ND | 4.92*+ | 21.00 | 9.98+ | 38.40 | 18.46*+ | 49.00 | 16.92*+ | 62.40* | 58.72*+ |
| **RS31** | 18.0 | 19.6*+ | 5.80 | 4.70*+ | 16.40 | 15.80 | 41.40 | 42.80 | 48.40 | 35.70 | 69.60* | 85.16*+ |
| **RS32** | 22.2 | 22.0*+ | 7.80* | 6.94*+ | 23.20 | 15.56 | 38.40 | 23.4*+ | 39.60 | 36.46 | 68.60* | 79.68*+ |
| **RS33** | 25.8 | 18.4 | 7.38* | 6.28* | 22.60* | 23.32* | 59.60* | 41.45 | 51.00 | 29.71 | 74.40* | 86.14*+ |
| **RS34** | 5.6* | ND | 6.22 | ND | 6.38* | ND | 16.78* | ND | 7.76* | ND | 41.20* | ND |
| **RS35** | 25.6 | 24.6*+ | 7.04* | 6.18 | ND | 21.34* | ND | 31.81+ | ND | 37.74 | ND | 92.32*+ |
| **RS37** | 14.4 | 20.4*+ | 7.04* | 6.02 | 19.00 | 22.24* | 32.00* | 38.72 | 38.40 | 41.44 | 73.60* | 89.64*+ |
| **RS38** | 13.0* | 15.0 | 7.28* | 5.64 | 19.80 | 16.68 | 28.60* | 34.44+ | 34.20 | 26.32+ | 67.40* | 74.48*+ |
| **RS39** | 23.8 | 13.0 | 6.58 | 5.42*+ | 20.00 | 13.70+ | 45.20 | 33.00+ | 48.00 | 36.20 | 77.40* | 94.78*+ |
| **RS40** | 18.4 | 27.8*+ | 5.72* | 5.02*+ | 24.00* | 22.28 | 39.40 | 34.14 | 35.80 | 34.54 | 68.00* | 76.26*+ |
| **RS41** | 18.4 | 18.2 | 6.24 | 6.12 | 19.10 | 20.62 | 35.80 | 38.06 | 38.80 | 36.42 | 68.80* | 91.50*+ |
| **RS42** | 17.2 | 15.2 | 6.04 | 4.78*+ | 12.56* | 13.34+ | 37.80 | 35.16 | 37.00 | 45.04 | 82.20 | 99.42*+ |
| **RS43** | 39.0 | 22.2* | 7.64* | 6.80*+ | 24.20* | 34.78*+ | 55.20* | 46.12 | 57.40* | 42.56 | 79.00* | 102.94*+ |
| **RS45** | 18.2 | 18.4 | 6.12 | 5.84 | 18.74 | 18.28 | 37.80 | 35.84 | 34.40 | 25.64+ | 55.60* | 66.24*+ |
| **RS46** | 16.6 | 18.8 | 6.04 | 5.82 | 19.96 | 23.34 | 29.40* | 32.32 | 31.40 | 23.26+ | 52.60* | 63.16*+ |
| **RS47** | 21.8 | 15.2 | 5.98 | 5.12*+ | 17.80 | 15.38 | 38.80 | 35.74 | 38.60 | 41.70 | 72.20* | 92.34*+ |
| **RS48(2017)_1** | 20.4 | NI | ND | NI | 25.00 | NI | 45.00 | NI | 30.60 | NI | 70.60* | NI |
| **RS48(2017)_2** | ND | NI | ND | NI | ND | NI | ND | NI | ND | NI | ND | NI |
| **RS48(2017)_3** | ND | NI | ND | NI | ND | NI | ND | NI | ND | NI | ND | NI |
| **RS48(2018)** | NI | 18.4 | NI | 5.68 | NI | 18.38 | NI | 45.76 | NI | 43.44 | NI | 86.28*+ |
| **RS49** | 11.8* | 19.6* | 5.94 | 5.24*+ | 15.26 | 19.22 | 34.00 | 33.20 | 30.80* | 37.36 | 74.00* | 91.04*+ |
| **RS50** | 15.0 | 20.2*+ | 5.12* | 5.12*+ | 12.48 | 20.32* | 45.40 | 48.04 | 33.40 | 44.10 | 83.80 | 102.16*+ |

^w^ *significantly different from V-C; +significantly different from T-C

^x^ Means in hatched boxes were compared to V-C and T-C through Mann-Whitney U test

^y^ Comparisons to T-C of all means through Mann-Whitney U test

^z^ Comparisons to V-C of all means through Mann-Whitney U test

FN: flower number; FS: flower size; FW: flower weight; LW: leaf weight; SW: stalk weight; PS: plant size; V-C: in vivo control; T-C: in vitro control; ND: not determined (less than 5 cuttings available); NI: not included
